# Di-μ-hydroxido-bis­[bromidodi-*p*-tolyl­tin(IV)]

**DOI:** 10.1107/S1600536809019758

**Published:** 2009-06-06

**Authors:** Kong Mun Lo, Seik Weng Ng

**Affiliations:** a Department of Chemistry, University of Malaya, 50603 Kuala Lumpur, Malaysia

## Abstract

The Sn atoms in the dinuclear title compound, [Sn_2_Br_2_(C_7_H_7_)_4_(OH)_2_], exist in distorted trigonal-bipyramidal BrSnC_2_O_2_ coordination geometries. Each of the two independent dinuclear mol­ecules comprising the asymmetric unit is disposed about a center of inversion. In the crystal, molecules are linked by an O—H⋯ hydrogen bond.

## Related literature

For other dihalo-di-*μ*-hydoxotetra­organylditins, see: Anacona *et al.* (2003 ▶); Barba *et al.* (2007 ▶); Puff *et al.* (1984 ▶).
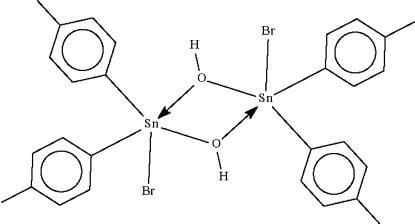


## Experimental

### 

#### Crystal data

[Sn_2_Br_2_(C_7_H_7_)_4_(OH)_2_]
*M* *_r_* = 795.72
Triclinic, 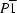

*a* = 10.9971 (3) Å
*b* = 11.5391 (3) Å
*c* = 12.1969 (3) Å
α = 77.092 (2)°
β = 86.552 (2)°
γ = 68.204 (2)°
*V* = 1400.34 (6) Å^3^
*Z* = 2
Mo *K*α radiation
μ = 4.66 mm^−1^
*T* = 100 K
0.35 × 0.05 × 0.05 mm

#### Data collection

Bruker SMART APEX diffractometer
Absorption correction: multi-scan (*SADABS*; Sheldrick, 1996 ▶) *T*_min_ = 0.292, *T*_max_ = 0.800
9026 measured reflections
4871 independent reflections
3367 reflections with *I* > 2σ(*I*)
*R*_int_ = 0.056

#### Refinement

*R*[*F* ^2^ > 2σ(*F* ^2^)] = 0.076
*wR*(*F* ^2^) = 0.227
*S* = 1.05
4871 reflections
311 parameters
180 restraints
H-atom parameters constrained
Δρ_max_ = 2.96 e Å^−3^
Δρ_min_ = −3.19 e Å^−3^

### 

Data collection: *APEX2* (Bruker, 2007 ▶); cell refinement: *SAINT* (Bruker, 2007 ▶); data reduction: *SAINT*; program(s) used to solve structure: *SHELXS97* (Sheldrick, 2008 ▶); program(s) used to refine structure: *SHELXL97* (Sheldrick, 2008 ▶); molecular graphics: *X-SEED* (Barbour, 2001 ▶); software used to prepare material for publication: *publCIF* (Westrip, 2009 ▶).

## Supplementary Material

Crystal structure: contains datablocks global, I. DOI: 10.1107/S1600536809019758/tk2460sup1.cif

Structure factors: contains datablocks I. DOI: 10.1107/S1600536809019758/tk2460Isup2.hkl

Additional supplementary materials:  crystallographic information; 3D view; checkCIF report

## Figures and Tables

**Table 1:** Hydrogen-bond geometry (Å, °)

*D*—H⋯*A*	*D*—H	H⋯*A*	*D*⋯*A*	*D*—H⋯*A*
O2—H2⋯Br1^i^	0.84	2.49	3.329 (8)	173

Symmetry code: (i) 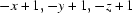.

**Fig. 1.**
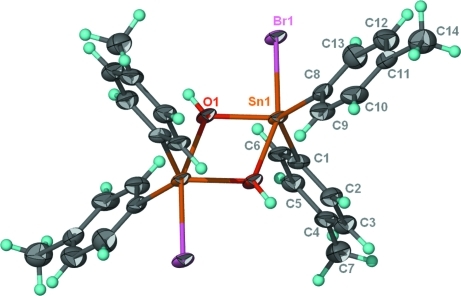
Thermal ellipsoid plot (Barbour, 2001) of one independent molecule of Sn2Br2(OH)2(C7H7)4 at the 70% probability level. Hydrogen atoms are drawn as spheres of arbitrary radius.

**Fig. 2.**
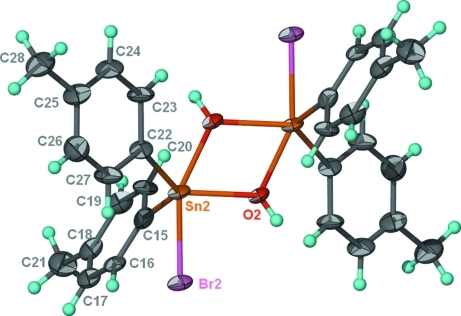
Thermal ellipsoid plot (Barbour, 2001) of the second independent molecule of Sn2Br2(OH)2(C7H7)4at the 70% probability level. Hydrogen atoms are drawn as spheres of arbitrary radius.

## supplementary crystallographic information

### Experimental

Di(*p*-tolyl)dimethyltin was synthesized by a Grignard reaction. This compound (3.37 g, 10 mmol) and pyridinium tribromide (3.19 g, 10 mmol) were heated in an ethanol/chloroform mixture for 1 hour. The solution was set aside for the growth of crystals. The organic reactant probably cleaved the two tin-methyl bonds to form di(*p*-tolyl)tin dibromide, which then underwent hydrolysis to the title compound.

### Refinement

Hydrogen atoms were placed at calculated positions (C–H 0.95–0.98 Å) and were treated as riding on their parent atoms, with *U*(H) set to 1.2–1.5 times *U*_eq_(C). The hydroxyl H-atom was similarly treated; O—H 0.84 Å and *U*(H) set to 1.2 times *U*_eq_(O).

The final difference Fourier map had a large peaks/deep holes at approximately 1 Å from Sn2 but was otherwise featureless.

The plate-like nature of the crystal, along with the presence of heavy atoms, adversedly affected the quality of the diffraction data.  As some of the displacement ellipsoids were rather elongated, the refinement strategy was to restrain the anisotropic displacement parameters of all carbon and oxygen atoms to be nearly isotropic.

### Crystal data

**Table:**

[Sn_2_Br_2_(C_7_H_7_)_4_(OH)_2_]	*Z* = 2
*M**_r_* = 795.72	*F*(000) = 768
Triclinic, *P*1	*D*_x_ = 1.887 Mg m^−^^3^
Hall symbol: -P 1	Mo *K*α radiation, λ = 0.71073 Å
*a* = 10.9971 (3) Å	Cell parameters from 3015 reflections
*b* = 11.5391 (3) Å	θ = 2.2–28.0°
*c* = 12.1969 (3) Å	µ = 4.66 mm^−^^1^
α = 77.092 (2)°	*T* = 100 K
β = 86.552 (2)°	Plate, colorless
γ = 68.204 (2)°	0.35 × 0.05 × 0.05 mm
*V* = 1400.34 (6) Å^3^	

### Data collection

**Table:**

Bruker SMART APEX diffractometer	4871 independent reflections
Radiation source: fine-focus sealed tube	3367 reflections with *I* > 2σ(*I*)
graphite	*R*_int_ = 0.056
ω scans	θ_max_ = 25.0°, θ_min_ = 1.7°
Absorption correction: multi-scan (*SADABS*; Sheldrick, 1996)	*h* = −13→13
*T*_min_ = 0.292, *T*_max_ = 0.800	*k* = −12→13
9026 measured reflections	*l* = −14→14

### Refinement

**Table:**

Refinement on *F*^2^	Primary atom site location: structure-invariant direct methods
Least-squares matrix: full	Secondary atom site location: difference Fourier map
*R*[*F*^2^ > 2σ(*F*^2^)] = 0.076	Hydrogen site location: inferred from neighbouring sites
*wR*(*F*^2^) = 0.227	H-atom parameters constrained
*S* = 1.05	*w* = 1/[σ^2^(*F*_o_^2^) + (0.1452*P*)^2^] where *P* = (*F*_o_^2^ + 2*F*_c_^2^)/3
4871 reflections	(Δ/σ)_max_ = 0.001
311 parameters	Δρ_max_ = 2.96 e Å^−^^3^
180 restraints	Δρ_min_ = −3.19 e Å^−^^3^

### Fractional atomic coordinates and isotropic or equivalent isotropic displacement parameters (Å^2^)

**Table:**

	*x*	*y*	*z*	*U*_iso_*/*U*_eq_	
Sn1	0.62921 (7)	0.55224 (9)	0.51125 (6)	0.0230 (3)	
Sn2	0.64941 (7)	0.38277 (9)	0.05546 (6)	0.0247 (3)	
Br1	0.68040 (11)	0.63918 (14)	0.67783 (9)	0.0280 (4)	
Br2	0.67660 (12)	0.23425 (14)	0.25580 (10)	0.0302 (4)	
O1	0.4708 (7)	0.5278 (8)	0.5909 (6)	0.0248 (19)	
H1	0.4458	0.5453	0.6538	0.030*	
O2	0.4555 (7)	0.4658 (8)	0.0890 (6)	0.0244 (19)	
H2	0.4206	0.4451	0.1496	0.029*	
C1	0.5776 (11)	0.7262 (12)	0.3905 (10)	0.024 (3)	
C2	0.6189 (12)	0.7250 (13)	0.2814 (10)	0.029 (3)	
H2a	0.6689	0.6469	0.2599	0.035*	
C3	0.5850 (12)	0.8423 (14)	0.2031 (11)	0.031 (3)	
H3	0.6153	0.8416	0.1285	0.038*	
C4	0.5107 (11)	0.9574 (13)	0.2283 (10)	0.029 (3)	
C5	0.4709 (12)	0.9557 (13)	0.3400 (10)	0.029 (3)	
H5	0.4197	1.0340	0.3606	0.034*	
C6	0.5041 (11)	0.8434 (13)	0.4206 (10)	0.028 (3)	
H6	0.4777	0.8449	0.4960	0.033*	
C7	0.4664 (13)	1.0812 (15)	0.1414 (11)	0.039 (3)	
H7A	0.5365	1.0817	0.0879	0.058*	
H7B	0.4458	1.1530	0.1786	0.058*	
H7C	0.3881	1.0895	0.1011	0.058*	
C8	0.8096 (11)	0.3956 (13)	0.5251 (10)	0.026 (3)	
C9	0.8241 (12)	0.2834 (13)	0.4902 (10)	0.031 (3)	
H9	0.7496	0.2743	0.4631	0.037*	
C10	0.9453 (13)	0.1859 (15)	0.4946 (11)	0.036 (3)	
H10	0.9535	0.1107	0.4702	0.043*	
C11	1.0547 (12)	0.1972 (14)	0.5344 (10)	0.031 (3)	
C12	1.0404 (13)	0.3050 (15)	0.5719 (12)	0.040 (4)	
H12	1.1143	0.3120	0.6025	0.048*	
C13	0.9190 (13)	0.4043 (16)	0.5658 (12)	0.041 (4)	
H13	0.9117	0.4792	0.5901	0.049*	
C14	1.1892 (13)	0.0913 (16)	0.5399 (13)	0.045 (4)	
H14A	1.2457	0.1205	0.4844	0.067*	
H14B	1.1802	0.0155	0.5234	0.067*	
H14C	1.2282	0.0698	0.6154	0.067*	
C15	0.7678 (11)	0.4819 (14)	0.0875 (10)	0.028 (3)	
C16	0.8608 (11)	0.4277 (12)	0.1749 (9)	0.023 (3)	
H16	0.8676	0.3492	0.2245	0.027*	
C17	0.9444 (12)	0.4905 (13)	0.1889 (10)	0.030 (3)	
H17	1.0081	0.4528	0.2486	0.035*	
C18	0.9376 (12)	0.6030 (14)	0.1205 (11)	0.032 (3)	
C19	0.8446 (12)	0.6556 (15)	0.0312 (11)	0.035 (3)	
H19	0.8385	0.7338	−0.0186	0.042*	
C20	0.7623 (12)	0.5951 (13)	0.0151 (10)	0.031 (3)	
H20	0.7010	0.6314	−0.0463	0.037*	
C21	1.0280 (13)	0.6689 (15)	0.1349 (12)	0.040 (4)	
H21A	1.0536	0.7054	0.0609	0.060*	
H21B	1.1063	0.6070	0.1783	0.060*	
H21C	0.9833	0.7373	0.1751	0.060*	
C22	0.7048 (11)	0.2359 (13)	−0.0370 (10)	0.027 (3)	
C23	0.6804 (12)	0.2653 (14)	−0.1532 (10)	0.030 (3)	
H23	0.6283	0.3495	−0.1909	0.036*	
C24	0.7342 (13)	0.1684 (14)	−0.2122 (10)	0.033 (3)	
H24	0.7131	0.1866	−0.2901	0.039*	
C25	0.8156 (13)	0.0490 (15)	−0.1629 (11)	0.035 (3)	
C26	0.8421 (13)	0.0196 (15)	−0.0484 (12)	0.037 (3)	
H26	0.8979	−0.0639	−0.0123	0.045*	
C27	0.7869 (12)	0.1125 (13)	0.0126 (10)	0.031 (3)	
H27	0.8053	0.0918	0.0912	0.037*	
C28	0.8804 (14)	−0.0516 (15)	−0.2323 (12)	0.041 (4)	
H28A	0.9194	−0.0147	−0.2987	0.061*	
H28B	0.8146	−0.0799	−0.2563	0.061*	
H28C	0.9490	−0.1248	−0.1866	0.061*	

### Atomic displacement parameters (Å^2^)

**Table:**

	*U*^11^	*U*^22^	*U*^33^	*U*^12^	*U*^13^	*U*^23^
Sn1	0.0242 (4)	0.0333 (6)	0.0114 (4)	−0.0063 (4)	0.0016 (3)	−0.0123 (4)
Sn2	0.0272 (5)	0.0359 (6)	0.0117 (4)	−0.0082 (4)	0.0011 (3)	−0.0127 (4)
Br1	0.0312 (6)	0.0416 (9)	0.0135 (6)	−0.0108 (6)	0.0014 (5)	−0.0155 (6)
Br2	0.0346 (7)	0.0402 (9)	0.0134 (6)	−0.0085 (6)	0.0002 (5)	−0.0104 (6)
O1	0.027 (4)	0.037 (5)	0.012 (4)	−0.009 (4)	0.005 (3)	−0.014 (4)
O2	0.024 (4)	0.034 (5)	0.014 (4)	−0.007 (3)	0.003 (3)	−0.012 (3)
C1	0.025 (5)	0.025 (6)	0.017 (5)	−0.002 (5)	−0.005 (4)	−0.004 (5)
C2	0.031 (6)	0.031 (7)	0.022 (6)	−0.008 (5)	−0.002 (4)	−0.005 (5)
C3	0.033 (6)	0.039 (7)	0.023 (6)	−0.010 (5)	0.003 (5)	−0.014 (5)
C4	0.029 (5)	0.036 (7)	0.022 (5)	−0.007 (5)	−0.005 (4)	−0.014 (5)
C5	0.029 (5)	0.034 (7)	0.024 (6)	−0.008 (5)	−0.001 (4)	−0.016 (5)
C6	0.029 (5)	0.036 (7)	0.016 (5)	−0.005 (5)	−0.001 (4)	−0.013 (5)
C7	0.038 (6)	0.046 (7)	0.030 (6)	−0.013 (5)	0.000 (5)	−0.009 (6)
C8	0.024 (5)	0.036 (7)	0.015 (5)	−0.002 (5)	0.004 (4)	−0.014 (5)
C9	0.033 (6)	0.033 (7)	0.024 (6)	−0.006 (5)	−0.004 (5)	−0.010 (5)
C10	0.043 (6)	0.042 (7)	0.026 (6)	−0.011 (5)	−0.002 (5)	−0.020 (5)
C11	0.031 (6)	0.040 (7)	0.018 (5)	−0.010 (5)	0.002 (4)	−0.005 (5)
C12	0.034 (6)	0.051 (8)	0.035 (6)	−0.009 (5)	−0.002 (5)	−0.018 (6)
C13	0.038 (6)	0.047 (8)	0.042 (7)	−0.015 (6)	0.003 (5)	−0.019 (6)
C14	0.042 (6)	0.046 (8)	0.040 (7)	−0.005 (6)	−0.001 (5)	−0.014 (6)
C15	0.023 (5)	0.040 (7)	0.024 (6)	−0.011 (5)	0.005 (4)	−0.018 (5)
C16	0.028 (5)	0.025 (6)	0.014 (5)	−0.005 (5)	0.001 (4)	−0.011 (5)
C17	0.030 (5)	0.039 (7)	0.021 (5)	−0.010 (5)	0.004 (4)	−0.017 (5)
C18	0.033 (6)	0.038 (7)	0.027 (6)	−0.008 (5)	0.009 (5)	−0.019 (5)
C19	0.037 (6)	0.041 (7)	0.027 (6)	−0.015 (5)	0.003 (5)	−0.008 (5)
C20	0.032 (6)	0.041 (7)	0.016 (5)	−0.004 (5)	−0.009 (4)	−0.011 (5)
C21	0.040 (6)	0.051 (8)	0.036 (6)	−0.020 (6)	−0.003 (5)	−0.016 (6)
C22	0.030 (5)	0.032 (7)	0.022 (5)	−0.007 (5)	−0.002 (4)	−0.018 (5)
C23	0.038 (6)	0.036 (7)	0.014 (5)	−0.010 (5)	−0.003 (4)	−0.009 (5)
C24	0.043 (6)	0.036 (7)	0.020 (5)	−0.007 (5)	−0.003 (5)	−0.019 (5)
C25	0.041 (6)	0.045 (7)	0.025 (6)	−0.014 (5)	0.000 (5)	−0.022 (5)
C26	0.034 (6)	0.039 (7)	0.037 (6)	−0.009 (5)	0.000 (5)	−0.012 (6)
C27	0.043 (6)	0.031 (7)	0.016 (5)	−0.008 (5)	−0.007 (5)	−0.009 (5)
C28	0.048 (6)	0.044 (7)	0.031 (6)	−0.013 (6)	0.005 (5)	−0.021 (6)

### Geometric parameters (Å, °)

**Table:**

Sn1—O1	2.024 (8)	C11—C14	1.523 (18)
Sn1—C8	2.115 (12)	C12—C13	1.39 (2)
Sn1—C1	2.111 (12)	C12—H12	0.9500
Sn1—O1^i^	2.248 (8)	C13—H13	0.9500
Sn1—Br1	2.6304 (14)	C14—H14A	0.9800
Sn2—O2	2.046 (8)	C14—H14B	0.9800
Sn2—C22	2.126 (13)	C14—H14C	0.9800
Sn2—C15	2.127 (13)	C15—C20	1.386 (19)
Sn2—O2^ii^	2.205 (8)	C15—C16	1.390 (16)
Sn2—Br2	2.6141 (15)	C16—C17	1.402 (18)
O1—Sn1^i^	2.248 (8)	C16—H16	0.9500
O1—H1	0.8400	C17—C18	1.357 (19)
O2—Sn2^ii^	2.205 (8)	C17—H17	0.9500
O2—H2	0.8400	C18—C19	1.406 (18)
C1—C2	1.381 (17)	C18—C21	1.496 (19)
C1—C6	1.411 (18)	C19—C20	1.378 (19)
C2—C3	1.405 (18)	C19—H19	0.9500
C2—H2a	0.9500	C20—H20	0.9500
C3—C4	1.368 (19)	C21—H21A	0.9800
C3—H3	0.9500	C21—H21B	0.9800
C4—C5	1.404 (17)	C21—H21C	0.9800
C4—C7	1.507 (19)	C22—C27	1.391 (18)
C5—C6	1.380 (18)	C22—C23	1.400 (16)
C5—H5	0.9500	C23—C24	1.390 (19)
C6—H6	0.9500	C23—H23	0.9500
C7—H7A	0.9800	C24—C25	1.357 (19)
C7—H7B	0.9800	C24—H24	0.9500
C7—H7C	0.9800	C25—C26	1.383 (19)
C8—C13	1.374 (18)	C25—C28	1.525 (19)
C8—C9	1.403 (19)	C26—C27	1.37 (2)
C9—C10	1.383 (18)	C26—H26	0.9500
C9—H9	0.9500	C27—H27	0.9500
C10—C11	1.384 (18)	C28—H28A	0.9800
C10—H10	0.9500	C28—H28B	0.9800
C11—C12	1.37 (2)	C28—H28C	0.9800
			
O1—Sn1—C8	119.8 (4)	C10—C11—C14	121.3 (14)
O1—Sn1—C1	112.0 (4)	C11—C12—C13	120.8 (13)
C8—Sn1—C1	126.5 (5)	C11—C12—H12	119.6
O1—Sn1—O1^i^	69.1 (3)	C13—C12—H12	119.6
C8—Sn1—O1^i^	94.3 (4)	C8—C13—C12	121.1 (15)
C1—Sn1—O1^i^	91.6 (4)	C8—C13—H13	119.5
O1—Sn1—Br1	90.9 (2)	C12—C13—H13	119.5
C8—Sn1—Br1	95.4 (3)	C11—C14—H14A	109.5
C1—Sn1—Br1	96.5 (3)	C11—C14—H14B	109.5
O1^i^—Sn1—Br1	160.0 (2)	H14A—C14—H14B	109.5
O2—Sn2—C22	118.3 (4)	C11—C14—H14C	109.5
O2—Sn2—C15	114.3 (4)	H14A—C14—H14C	109.5
C22—Sn2—C15	126.0 (5)	H14B—C14—H14C	109.5
O2—Sn2—O2^ii^	69.2 (4)	C20—C15—C16	119.0 (12)
C22—Sn2—O2^ii^	93.8 (4)	C20—C15—Sn2	120.5 (9)
C15—Sn2—O2^ii^	93.7 (4)	C16—C15—Sn2	120.3 (10)
O2—Sn2—Br2	87.4 (2)	C15—C16—C17	119.1 (12)
C22—Sn2—Br2	96.9 (3)	C15—C16—H16	120.4
C15—Sn2—Br2	96.7 (3)	C17—C16—H16	120.4
O2^ii^—Sn2—Br2	156.6 (2)	C18—C17—C16	122.4 (12)
Sn1—O1—Sn1^i^	110.9 (3)	C18—C17—H17	118.8
Sn1—O1—H1	124.6	C16—C17—H17	118.8
Sn1^i^—O1—H1	124.6	C17—C18—C19	117.8 (13)
Sn2—O2—Sn2^ii^	110.8 (4)	C17—C18—C21	122.2 (12)
Sn2—O2—H2	124.6	C19—C18—C21	119.9 (13)
Sn2^ii^—O2—H2	124.6	C20—C19—C18	120.8 (14)
C2—C1—C6	119.9 (11)	C20—C19—H19	119.6
C2—C1—Sn1	119.4 (10)	C18—C19—H19	119.6
C6—C1—Sn1	120.7 (9)	C19—C20—C15	120.8 (11)
C1—C2—C3	118.2 (13)	C19—C20—H20	119.6
C1—C2—H2a	120.9	C15—C20—H20	119.6
C3—C2—H2a	120.9	C18—C21—H21A	109.5
C4—C3—C2	123.6 (12)	C18—C21—H21B	109.5
C4—C3—H3	118.2	H21A—C21—H21B	109.5
C2—C3—H3	118.2	C18—C21—H21C	109.5
C3—C4—C5	116.9 (13)	H21A—C21—H21C	109.5
C3—C4—C7	123.0 (12)	H21B—C21—H21C	109.5
C5—C4—C7	120.0 (13)	C27—C22—C23	118.1 (12)
C6—C5—C4	121.7 (13)	C27—C22—Sn2	120.1 (9)
C6—C5—H5	119.2	C23—C22—Sn2	120.8 (10)
C4—C5—H5	119.2	C24—C23—C22	118.5 (12)
C5—C6—C1	119.7 (11)	C24—C23—H23	120.8
C5—C6—H6	120.2	C22—C23—H23	120.8
C1—C6—H6	120.2	C25—C24—C23	122.6 (12)
C4—C7—H7A	109.5	C25—C24—H24	118.7
C4—C7—H7B	109.5	C23—C24—H24	118.7
H7A—C7—H7B	109.5	C24—C25—C26	119.2 (13)
C4—C7—H7C	109.5	C24—C25—C28	120.9 (12)
H7A—C7—H7C	109.5	C26—C25—C28	119.8 (13)
H7B—C7—H7C	109.5	C27—C26—C25	119.3 (13)
C13—C8—C9	117.9 (12)	C27—C26—H26	120.4
C13—C8—Sn1	119.5 (11)	C25—C26—H26	120.4
C9—C8—Sn1	122.6 (9)	C26—C27—C22	122.2 (12)
C10—C9—C8	120.9 (12)	C26—C27—H27	118.9
C10—C9—H9	119.5	C22—C27—H27	118.9
C8—C9—H9	119.5	C25—C28—H28A	109.5
C11—C10—C9	120.3 (14)	C25—C28—H28B	109.5
C11—C10—H10	119.8	H28A—C28—H28B	109.5
C9—C10—H10	119.8	C25—C28—H28C	109.5
C12—C11—C10	119.0 (12)	H28A—C28—H28C	109.5
C12—C11—C14	119.6 (12)	H28B—C28—H28C	109.5
			
C8—Sn1—O1—Sn1^i^	−82.8 (5)	C14—C11—C12—C13	178.7 (13)
C1—Sn1—O1—Sn1^i^	83.0 (5)	C9—C8—C13—C12	0(2)
O1^i^—Sn1—O1—Sn1^i^	0.0	Sn1—C8—C13—C12	−177.5 (11)
Br1—Sn1—O1—Sn1^i^	−179.6 (3)	C11—C12—C13—C8	2(2)
C22—Sn2—O2—Sn2^ii^	−82.9 (6)	O2—Sn2—C15—C20	−74.5 (10)
C15—Sn2—O2—Sn2^ii^	84.5 (5)	C22—Sn2—C15—C20	91.8 (11)
O2^ii^—Sn2—O2—Sn2^ii^	0.0	O2^ii^—Sn2—C15—C20	−5.6 (10)
Br2—Sn2—O2—Sn2^ii^	−179.4 (3)	Br2—Sn2—C15—C20	−164.6 (10)
O1—Sn1—C1—C2	−127.0 (9)	O2—Sn2—C15—C16	111.9 (10)
C8—Sn1—C1—C2	37.7 (12)	C22—Sn2—C15—C16	−81.8 (11)
O1^i^—Sn1—C1—C2	−58.9 (10)	O2^ii^—Sn2—C15—C16	−179.3 (9)
Br1—Sn1—C1—C2	139.4 (9)	Br2—Sn2—C15—C16	21.8 (10)
O1—Sn1—C1—C6	54.4 (11)	C20—C15—C16—C17	1.4 (18)
C8—Sn1—C1—C6	−140.9 (9)	Sn2—C15—C16—C17	175.2 (9)
O1^i^—Sn1—C1—C6	122.5 (10)	C15—C16—C17—C18	0.3 (19)
Br1—Sn1—C1—C6	−39.3 (10)	C16—C17—C18—C19	−1.4 (19)
C6—C1—C2—C3	−0.4 (18)	C16—C17—C18—C21	−179.3 (12)
Sn1—C1—C2—C3	−179.0 (9)	C17—C18—C19—C20	1(2)
C1—C2—C3—C4	−1.6 (19)	C21—C18—C19—C20	178.7 (13)
C2—C3—C4—C5	2.0 (19)	C18—C19—C20—C15	1(2)
C2—C3—C4—C7	−175.1 (12)	C16—C15—C20—C19	−2.0 (19)
C3—C4—C5—C6	−0.4 (19)	Sn2—C15—C20—C19	−175.8 (10)
C7—C4—C5—C6	176.8 (11)	O2—Sn2—C22—C27	−116.7 (10)
C4—C5—C6—C1	−1.4 (18)	C15—Sn2—C22—C27	77.5 (12)
C2—C1—C6—C5	1.8 (18)	O2^ii^—Sn2—C22—C27	174.9 (10)
Sn1—C1—C6—C5	−179.6 (9)	Br2—Sn2—C22—C27	−25.9 (10)
O1—Sn1—C8—C13	−123.0 (10)	O2—Sn2—C22—C23	75.3 (11)
C1—Sn1—C8—C13	73.4 (12)	C15—Sn2—C22—C23	−90.5 (11)
O1^i^—Sn1—C8—C13	168.7 (10)	O2^ii^—Sn2—C22—C23	6.9 (10)
Br1—Sn1—C8—C13	−28.8 (11)	Br2—Sn2—C22—C23	166.0 (10)
O1—Sn1—C8—C9	59.6 (11)	C27—C22—C23—C24	3.0 (19)
C1—Sn1—C8—C9	−104.0 (11)	Sn2—C22—C23—C24	171.3 (10)
O1^i^—Sn1—C8—C9	−8.8 (10)	C22—C23—C24—C25	−4(2)
Br1—Sn1—C8—C9	153.7 (10)	C23—C24—C25—C26	3(2)
C13—C8—C9—C10	−1.3 (19)	C23—C24—C25—C28	−175.5 (13)
Sn1—C8—C9—C10	176.3 (10)	C24—C25—C26—C27	−1(2)
C8—C9—C10—C11	0(2)	C28—C25—C26—C27	177.6 (13)
C9—C10—C11—C12	2(2)	C25—C26—C27—C22	0(2)
C9—C10—C11—C14	−179.9 (12)	C23—C22—C27—C26	−1(2)
C10—C11—C12—C13	−3(2)	Sn2—C22—C27—C26	−169.4 (11)

Symmetry codes: (i) −*x*+1, −*y*+1, −*z*+1; (ii) −*x*+1, −*y*+1, −*z*.

### Hydrogen-bond geometry (Å, °)

**Table:**

*D*—H···*A*	*D*—H	H···*A*	*D*···*A*	*D*—H···*A*
O2—H2···Br1^i^	0.84	2.49	3.329 (8)	173

Symmetry codes: (i) −*x*+1, −*y*+1, −*z*+1.
